# HSP90B1-mediated plasma membrane localization of GLUT1 promotes radioresistance of glioblastomas

**DOI:** 10.7555/JBR.37.20220234

**Published:** 2023-09-28

**Authors:** Yanhui Li, Yuqian Ge, Mengjie Zhao, Fangshu Ding, Xiuxing Wang, Zhumei Shi, Xin Ge, Xiefeng Wang, Xu Qian

**Affiliations:** 1 Department of Nutrition and Food Hygiene, Center for Global Health, School of Public Health, Nanjing Medical University, Nanjing, Jiangsu 211166, China; 2 Institute for Brain Tumors, Jiangsu Key Lab of Cancer Biomarkers, Prevention and Treatment, Jiangsu Collaborative Innovation Center for Cancer Personalized Medicine, Nanjing Medical University, Nanjing, Jiangsu 211166, China; 3 Department of Neuro-Psychiatric Institute, the Affiliated Brain Hospital of Nanjing Medical University, Nanjing, Jiangsu 210029, China; 4 National Health Commission Key Laboratory of Antibody Technologies, Nanjing Medical University, Nanjing, Jiangsu 211166, China; 5 Department of Cell Biology, School of Basic Medical Sciences, Nanjing Medical University, Nanjing, Jiangsu 211166, China; 6 Department of Neurosurgery, the First Affiliated Hospital of Nanjing Medical University, Nanjing, Jiangsu 210029, China

**Keywords:** HSP90B1, glycolysis, GLUT1, glioblastoma, radiotherapy

## Abstract

Ionizing radiation is a popular and effective treatment option for glioblastoma (GBM). However, resistance to radiation therapy inevitably occurs during treatment. It is urgent to investigate the mechanisms of radioresistance in GBM and to find ways to improve radiosensitivity. Here, we found that heat shock protein 90 beta family member 1 (HSP90B1) was significantly upregulated in radioresistant GBM cell lines. More importantly, HSP90B1 promoted the localization of glucose transporter type 1, a key rate-limiting factor of glycolysis, on the plasma membrane, which in turn enhanced glycolytic activity and subsequently tumor growth and radioresistance of GBM cells. These findings imply that targeting HSP90B1 may effectively improve the efficacy of radiotherapy for GBM patients, a potential new approach to the treatment of glioblastoma.

## Introduction

Glioblastoma (GBM) is the most aggressive primary brain malignancy in adults ^[ 1] ^. With a median survival of approximately 15 months, its overall survival rates have not improved remarkably in the past 20 years ^[ 2] ^. Radiotherapy is widely used as a standard adjuvant treatment modality for GBM patients. It is also the primary treatment for eliminating highly proliferating tumor cells in inoperable GBMs ^[ 3] ^. However, resistance to radiotherapy inevitably occurs ^[ 4] ^. Signaling pathways, including AKT, Notch, Wnt/β-catenin, ATM/Chk2/p53, STAT3, and Hedgehog, as well as glioma stem cells (GSCs) have been shown to affect the radiation resistance of GBMs ^[ 5] ^. Alterations in the microenvironment, such as hypoxia, are also associated with radiation therapy resistance and poor prognosis of brain tumors ^[ 6] ^. Although considerable basic and clinical efforts have been invested in developing highly effective radiosensitizers that may improve the efficacy of radiotherapy, therapeutic avenues remain limited ^[ 7] ^. Therefore, understanding the mechanisms underlying radioresistance and exploring new strategies to improve patient prognosis are imperative.

Tumor cells, under aerobic conditions, control metabolism to favor aerobic glycolysis over oxidative phosphorylation to produce ATP, which is known as the Warburg effect ^[ 8] ^. This reprogramming of metabolism allows cancer cells to produce the biological raw materials needed for cell proliferation ^[ 9] ^. The Warburg effect is particularly important in brain tumors, because the brain is mostly fueled by glucose ^[ 10] ^. The first step in glycolysis, before any modification of the glucose molecule occurs, is glucose translocation from the extracellular to the cytoplasmic matrix ^[ 11] ^. This process is mediated by a family of unidirectional transporter proteins that are collectively referred to as the glucose transporter protein (GLUT) family. The major GLUT in GBMs is GLUT1, encoded by *SLC2A1*, whose availability is a rate-limiting step in the glycolytic process ^[ 12] ^. GLUT1 has been shown to be present at high levels in a variety of malignancies, including breast cancer ^[ 13] ^, lung cancer ^[ 14]^ and GBM ^[ 15] ^, and to be associated with poor cancer prognosis. Therefore, the targeted regulation of GLUT1 may be a key node in GBM treatment.

Heat shock protein 90 beta family member 1 (HSP90B1), also known as GP96, is a member of the heat shock protein 90 family and is mainly restricted to the endoplasmic reticulum and melanosomes, serving to stabilize and fold other proteins ^[ 16] ^. HSP90B1 expression is known to be associated with multiple pathogenic states, including tumorigenesis. More importantly, its high expression was shown to reduce the efficacy of radiotherapy for head and neck tumors and lead to poor prognosis ^[ 17] ^. However, little is known about the molecular mechanisms underlying HSP90B1-induced resistance to radiotherapy in GBM tumor cells.

In the current study, we revealed that HSP90B1 played an important role in developing radioresistance by promoting membrane localization of GLUT1 and subsequently leading to an activated glycolysis in GBM tumor cells.

## Materials and methods

### Antibodies

Alexa Fluor 594 anti-rabbit IgG (Cat. #SA00013-4, 1:300) and the antibodies against HSP90B1 (Cat. #14700-1-AP, 1:1000), ATP1A1 (Cat. #14418-1-AP, 1:1000), GAPDH (Cat. #60004-1-Ig, 1:2000), caspase-7 (Cat. #27155-1-AP, 1:1000), caspase-8 (Cat. #66093-1-Ig, 1:1000), and β-actin (Cat. #66009-1-Ig, 1:2000) were purchased from Proteintech (Wuhan, Hubei, China). The antibodies against γ-H2AX (9718S, 1:1000), caspase-3 (Cat. #9662S, 1:1000), caspase-9 (Cat. #9504S, 1:1000), and PARP (Cat. #9542S, 1:1000) were purchased from Cell Signaling Technology (Boston, MA, USA). The antibody against Flag was from Sigma-Aldrich (Shanghai, China). The antibody against GLUT1 (Cat. #ab115730, 1:1000) was from Abcam (Cambridge, UK). Anti-mouse secondary antibodies (Cat. #sc-2005, 1:2000), anti-rabbit secondary antibodies (Cat. #sc-2004, 1:2000) and GLUT1 siRNA (Cat. #sc-35 493) were purchased from Santa Cruz Biotechnology (Santa Cruz, CA, USA). Dulbecco's modified Eagle's medium (DMEM) was purchased from Gibco (Shanghai, China). Polyjet reagent was purchased from SignaGen (Rockville, MD, USA).

### Cell culture

Three GBM cell lines (U251, U87 and LN18) and human embryonic kidney 293T cells were cultured in DMEM supplemented with 10% fetal bovine serum (Hyclone, Logan, UT, USA) plus 1% penicillin/streptomycin. MGG8 cells, one type of GSCs, were cultured in Neurobasal medium (Life Technologies, Gaithersburg, MD, USA) supplemented with B27, L-glutamine, sodium pyruvate, basic fibroblast growth factor (10 ng/mL) and EGF (10 ng/mL, R&D Systems, Minneapolis, MN, USA). To generate stable cell lines for gene expression, cells were transduced with a lentivirus carrying the *HSP90B1* shRNA plasmid and screened by puromycin.

### DNA construction and transduction

Polymerase chain reaction (PCR)-amplified human HSP90B1 and GLUT1 were cloned into pcDNA3.1/hygro(+)-Flag vector. For lentivirus packaging, PCR-amplified *HSP90B1* shRNA was cloned into pLVX-puro vector and the lentivirus was packaged in 293T cells and the transduction procedure was performed as previously described ^[ 18] ^. For plasmid transfection, cells were plated at a density of 4 × 10 ^5^ per 60-mm dish or 1 × 10 ^5^ per well of a 6-well plate 18 h before transfection.

### Irradiation of cells and the establishment of radioresistant cell lines

Cells were irradiated using a cabinet X-ray system (RS2000 Pro, Rad Source Technologies, GA, USA). The radioresistant cell lines were developed from the respective GBM cell lines (U251, U87 and LN18). Cells (1 × 10 ^6^) were started to receive 5 Gray (Gy) of radiation. After one week's recovery, the cells were repeatedly receiving 5 Gy of radiation. During this period, U251 cells finally received a total radiation dose of 60 Gy, while U87 and LN18 cells finally received a total radiation dose of 100 Gy. The radioresistance of the established cell lines was validated *in vitro* through the colony formation assay and DNA damage detection. Cells were then further maintained at a dose of 5 Gy per week.

### RNA-seq

Total RNA samples from U251, U87, LN18, and the respective radioresistant cell lines were subjected to HiSeq RNA-Seq assays performed by BGI Tech Solutions (Shenzhen, Guangdong, China). Each sample contained the pooled RNA from three biological replicates of 1 × 10 ^7^ cells. Gene expression levels were quantified by the RSEM software package. We corrected for *P*-values using Bonferroni's method. Also, false positive and false negative errors were corrected by the FDR method. We used *P* < 0.05, FDR < 0.001, and |log _2_FC| ≥ 1 as the default threshold to determine the significance of gene expression differences. RNA-Seq data were deposited in the NCBI's Gene Expression Omnibus database (GEO GSE206917).

### RNA extraction and quantitative real-time PCR

Total RNA was purified using TRIzol reagent and was reversely transcribed into cDNA using the MonScript RTⅢ Super Mix with dsDNase (Monad, Suzhou, Jiangsu, China) following the manufacturer's instructions. Quantitative real-time PCR (qRT-PCR) analyses were performed by MonAmp Fast SYBR Green qPCR Mix (Monad) on an LC96 Real-Time PCR Detection System (Roche, Basel, Switzerland). Primer sequences used for qRT-PCR are listed in ***Supplementary Table 1*** (available online).

### Colony formation assay

The designated cells were seeded in 6-well plates at 500, 1000, 2000, 4000, 6000, and 8000 numbers and given 0, 2, 4, 6, 8, or 10 Gy of X-ray irradiation after 24 h, followed by incubation in the incubator for 14 days. The medium was replaced with fresh ones every two or three days. For GBM cells, colonies were fixed with methanol and stained with 0.5% crystal violet for 15 min. For GSC cells, colonies were incubated with nitroblue tetrazolium chloride dye overnight. Colonies (> 50 cells) were scored and counted under the microscope. Survival curves were fitted by nonlinear regression analysis based on a multi-target single-impact model.

### Apoptosis analysis

Apoptotic cells were measured by Annexin V-FITC/PI Apoptosis Detection Kit (Vazyme, Nanjing, Jiangsu, China). Briefly, cells were incubated with 5 μL Annexin V-FITC and 5 μL PI Staining solution for 10 min at room temperature in the dark at 24 h after treatment with or without 5-Gy irradiation.

For the terminal deoxynucleotidyl transferase-mediated deoxyuridine triphosphate nick end labeling (TUNEL) assay, mouse tumor tissues were sectioned at 4-μm thickness. Apoptotic cells were counted using the TUNEL BrightGreen Apoptosis Detection Kit (Vazyme) according to the manufacturer's instructions.

### Detection of intracellular reactive oxygen species (ROS)

Intracellular ROS level was assessed by the fluorescence probe DCFDA (Abcam) according to the manufacturer's instructions. Briefly, cells were incubated with 25 μmol/L DCFDA-containing culture media for 30 min at 37 ℃ in the dark at 4 h after treatment with or without 5-Gy irradiation. Green fluorescence was analyzed by flow cytometry.

### Glucose uptake assay

A glucose uptake assay kit (Promega, J1342, MA, USA) was used following the manufacturer's instructions. Briefly, 1 × 10 ^4^ GBM cells and 5 × 10 ^3^ GSC cells were seeded into 96-well plates and grew for 12 h. The cells were washed twice with PBS and incubated with 1 mmol/L of 2-deoxy-D-glucose (2-DG) for 10 min at 37 ℃. The uptake was terminated by the addition of an acid detergent solution (stop buffer), and a neutralization buffer was then added to neutralize the acid. The 2-Deoxy-D-glucose 6-phosphate (2-DG6P) detection reagent containing glucose-6-phosphate dehydrogenase, NADP ^+^, reductase, recombinant luciferase, and proluciferin substrate was added to the sample wells. The plate was incubated for 1 h at 25 ℃ and luminescence intensity was read on a luminometer with 0.3–1 s integration. The glucose uptake level was normalized according to the cell number counted in a duplicate sample.

### Lactate production assay

A lactate production assay kit (Promega, J5022) was used following the manufacturer's instructions. Briefly, the attached GBM cells (1 × 10 ^4^) and GSC cells (5 × 10 ^3^) seeded in 96-well plates were replaced with 200 μL serum-free DMEM or Neurobasal medium, and 6 h later, the culture medium was collected for measurements of lactate production.

### Glucose consumption assay

Glucose consumption was measured according to the published procedures with slight modifications ^[ 19] ^. In brief, GBM cells (1 × 10 ^6^) were seeded in 60 mm dishes. The medium was replaced with 3 mL serum-free DMEM, and after 16 h, the medium was collected to measure glucose consumption.

### Assessment of extracellular acidification rate (ECAR)

The ECAR was determined by the Seahorse XF96 extracellular flux analyzer (Agilent, Palo Alto, CA, USA). Cells (1 × 10 ^4^) were seeded 24 h before the assay. The XF Glycolysis Stress Test Kit was used to measure the glycolytic capacity. Glucose, oligomycin and 2-DG were diluted with the XF media and loaded into the cartridge to achieve final concentrations of 10, 20, and 50 mmol/L, respectively. ECAR was determined according to the manufacturer's instructions. The XF Cell Mito Stress Test Kit was used to measure cellular mitochondrial function.

### Immunoprecipitation and immunoblotting analyses

The cell lysis buffer was prepared with 50 mmol/L Tris-HCl (pH 7.5), 0.1% SDS, 1% Triton X-100, 150 mmol/L NaCl, 1 mmol/L dithiothreitol, 0.5 mmol/L EDTA, 1% proteinase inhibitor cocktail, 100 mmol/L sodium orthovanadate, 100 mmol/L sodium pyrophosphate, and 1 mmol/L sodium fluoride. The cultured cells were washed with cold PBS on ice three times before lysis. Then, the cell lysis buffer was added, and the cells were scraped from dishes and incubated for 15 min on ice for complete cell lysis. The samples were centrifuged at 4 ℃ to collect the supernatant. Corresponding antibodies were used for immunoprecipitation or immunoblot analyses.

### Immunofluorescence staining

GLUT1 localization was determined by immunofluorescence staining based on a laboratory and antibody recommended protocol. Briefly, cells were cultured on a 35 mm dish with 20 mm bottom wells. To label the plasma membrane of cells, GBM cells were incubated in 5.0 µg/mL wheat germ agglutinin (WGA) conjugates (Invitrogen, W6748, Carlsbad, CA, USA) for 10 min at 37 ℃. The cells were washed with ice-cold PBS three times, fixed with 4% paraformaldehyde in PBS for 20 min at 37 ℃, and incubated with 0.5% TritonX-100/PBS-Tween for 20 min and 1% BSA/PBS-Tween to permeabilize the cells and to block nonspecific protein-protein interactions. The cells were incubated with an anti-GLUT1 antibody (1:200 dilution) overnight at 4 ℃. The cells were washed with PBS and incubated with Alexa Fluor 594 anti-rabbit IgG (red, 1:500 dilution) for 2 h in the dark. The cells were then counterstained with DAPI and mounted using ProLong Gold antifade reagent (Beyotime, P0131, Shanghai, China). Images were obtained with an LSM700 laser confocal microscope.

### Plasma membrane fractionation

GBM cells (5 × 10 ^8^) were cultured on 15 cm dishes. For obtaining plasma membranes, GBM cells were collected into 10 mL of ice-cold HES buffer (250 mmol/L sucrose, 1 mmol/L EDTA, 1 mmol/L phenylmethylsulfonyl fluoride [PMSF], 1 μmol/L pepstatin, 1 μmol/L aprotinin, 1 μmol/L leupeptin, and 20 mmol/L HEPES, pH 7.4) and subsequently homogenized with 20 strokes in a glass Dounce homogenizer at 4 ℃. After centrifugation at 1000 *g* for 3 min at 4 ℃ to remove large cell debris and unbroken cells, the supernatant was then centrifuged at 245000 *g* for 90 min at 4 ℃ to yield a pellet of total cellular membranes and a supernatant representing the cytosolic fraction. The resulting pellet representing the total cellular membrane fraction was resuspended in HES buffer before use. The samples were incubated on ice for 5 min and centrifuged at 1000 *g* for 5 min at 4 ℃; an upper-phase solution containing plasma membrane proteins was collected and transferred to new tubes. The resulting upper-phase solution was diluted with 5× volume of water, followed by centrifuge at 15000 *g* for 30 min at 4 ℃ and then analyzed by immunoblotting.

### Mice and bioluminescent imaging

The use of athymic nude mice was approved by the Institutional Review Board of Nanjing Medical University (Nanjing, Jiangsu, China). U87-luciferase cells were cultured in DMEM medium supplemented with 10% fetal bovine serum. MGG8-luciferase cells were cultured in Neurobasal medium supplemented with B27, L-glutamine, sodium pyruvate, basic fibroblast growth factor (10 ng/mL) and EGF (10 ng/mL). All cells were incubated at 37°C incubator. U87 (1 × 10 ^6^) or MGG8 (1 × 10 ^5^) luciferase cells were injected into 4-week-old female mice. U87 nude mice were divided into six groups and MGG8 nude mice were divided into four groups with seven mice in each group. All mice were fed ordinary feed and drinking water by Nanjing Medical University Laboratory Animal Center. Mice received 5-Gy irradiation for three consecutive days using the X-ray system RS2000 Pro (Rad Source, Buford, GA, USA). To examine tumor volume, the animals were intraperitoneally injected with luciferin, anesthetized with isoflurane inhalation, and subjected to bioluminescence imaging with IVIS Spectrum System (PerkinElmer, Waltham, MA, USA). Mice were maintained until neurological signs were apparent, at which point they were sacrificed. The mouse brains were harvested, fixed in 4% formaldehyde, and embedded in paraffin.

### Immunohistochemical staining

Paraffin-embedded tissue sections were dewaxed, hydrated, and antigen repaired. They were then incubated with antibodies against γ-H2AX at a dilution of 1:100 overnight at 4 ℃, followed by secondary antibody incubation and DAB staining, and finally sealed after dehydration and transparency.

### Statistical analysis

Statistical analyses were conducted with a two-tailed unpaired Student's *t*-test comparing two independent sample groups and a one-way ANOVA test comparing more than two groups unless specifically indicated. Kaplan-Meier survival curves were generated using Prism software and statistical significance was assessed between groups using a log-rank test. All data are represented as the mean ± standard deviation of three independent experiments/samples unless otherwise specified. Differences in means were considered statistically significant at *P* < 0.05. Analyses were performed using the Microsoft Excel.

## Results

### HSP90B1 is an intrinsic predictor for the prognosis of irradiation therapy in GBM patients

To investigate the altered events in radioresistant GBM cells, we established radioresistant cells by exposing GBM parental cells, including U251, U87, and LN18, to 5 Gy of ionizing radiation (IR) weekly, to be U251-radioresistant cells (U251-RR), U87-radioresistant cells (U87-RR), and LN18-radioresistant cells (LN18-RR), respectively ( ***Supplementary Fig. 1A ***, available online).

**Figure 1:**
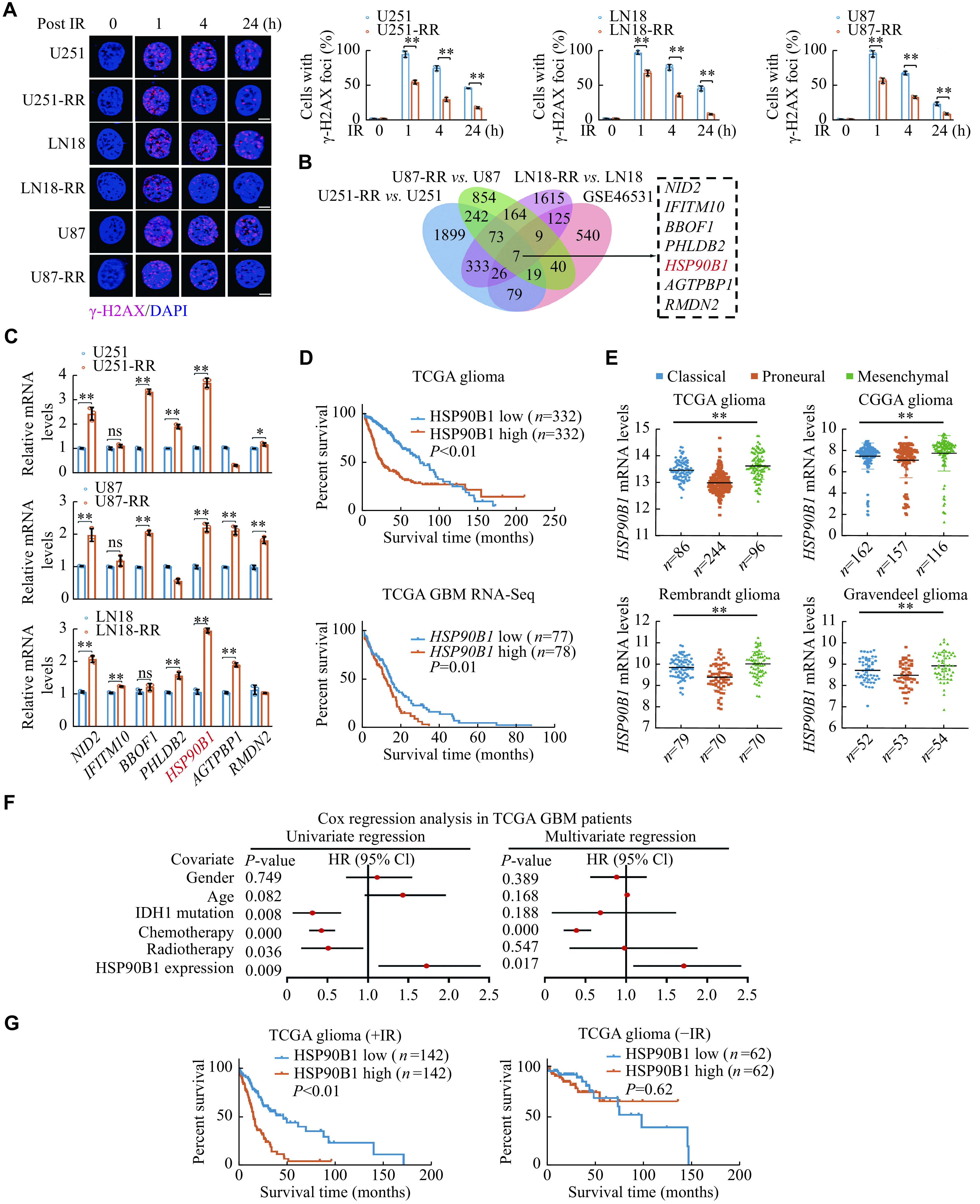
HSP90B1 is an intrinsic predictor for the prognosis of irradiation therapy in GBM patients.

To confirm the development of radioresistance, we treated GBM cells with different irradiation doses. After analyzing with a single-click multi-target model, the radioresistant cell lines showed an enhanced ability of colony formation ( ***Supplementary Fig. 1B ***, available online). The cell survival rates for U251 and U251-RR at a radiotherapy dose of 4 Gy were 0.12 and 0.34, respectively. Other GBM cells showed similar results. Furthermore, the accelerated DNA damage repair as evidenced by reduced levels of γ-H2AX ( ***Supplementary Fig. 1C ***, available online) and short-lived γ-H2AX positive foci ( ***Fig. 1A***) upon radiation challenge confirmed the success of radioresistant cell line establishment.

RNA-seq analyses were performed to investigate the altered genes that are differentially expressed in RR, compared with parental GBM cells. Integrated analyses, including our RNA-seq data and published data obtained from GSCs that were intrinsically resistant to radiation (GSE46531), suggested that seven genes were upregulated in RR cells, including *NID2*, *IFITM10*, *BBOF1*, *PHLDB2*, *HSP90B1*, *AGTPBP1*, and *RMDN2* ( ***Fig. 1B***). Further validation conducted by qPCR analyses demonstrated that, among these genes, expression levels of *NID2* and *HSP90B1*were generally increased in all the tested RR GBM cells, compared with their corresponding parental counterparts ( ***Fig. 1C***). No correlation between *NID2* expression with survival of GBMs or low-grade gliomas were observed in TCGA, a database that is well-accepted in glioma research ( ***Supplementary Fig. 2A ***, available online).

We next focused on HSP90B1 expression with glioma outcomes. By analyzing public databases, including TCGA, CGGA, Rembrandt ^[ 20] ^, and Gravendeel ^[ 21] ^, we found that the high expression level of HSP90B1 was associated with the reduced overall survival rate of glioma patients ( ***Fig. 1D*** and ***Supplementary Fig. 2B*** [available online]). In addition, HSP90B1 expression levels were significantly higher in GBMs (WHO grade Ⅳ) than those in gliomas of lower grades (including grades Ⅱ and Ⅲ), which has been suggested to be benefited from IR-based therapies ( ***Supplementary Fig. 2C ***, available online). Among GBMs, the *HSP90B1* expression level was greatly increased in the mesenchymal subtype, which is resistant to IR therapy, compared with the classical and proneural subtypes ( ***Fig. 1E***). We next performed COX analysis for information on GBM patients in the TCGA and CGGA databases. Multivariate analysis showed that *HSP90B1* expression was negatively associated with the survival of GBM patients in both TCGA (hazard ratio [HR], 1.624; *P* = 0.017; ***Fig. 1F***) and CGGA databases (HR, 1.321; *P* = 0.027; ***Supplementary Fig. 2D ***, available online). HSP90B1 expression did not segregate glioma patients on survival benefits when they did not receive IR treatment ( ***Fig. 1G***, left). However, when receiving IR treatment, GBM patients with a higher HSP90B1 expression level exhibited a shorter survival rate than those with a lower HSP90B1 expression level in TCGA databases ( ***Fig. 1G***, right). Together, these data suggested that HSP90B1 expression predicted the response of GBM patients to irradiation therapy.

### HSP90B1 inhibits the sensitivity of GBM cells to radiation therapy

**Figure 2:**
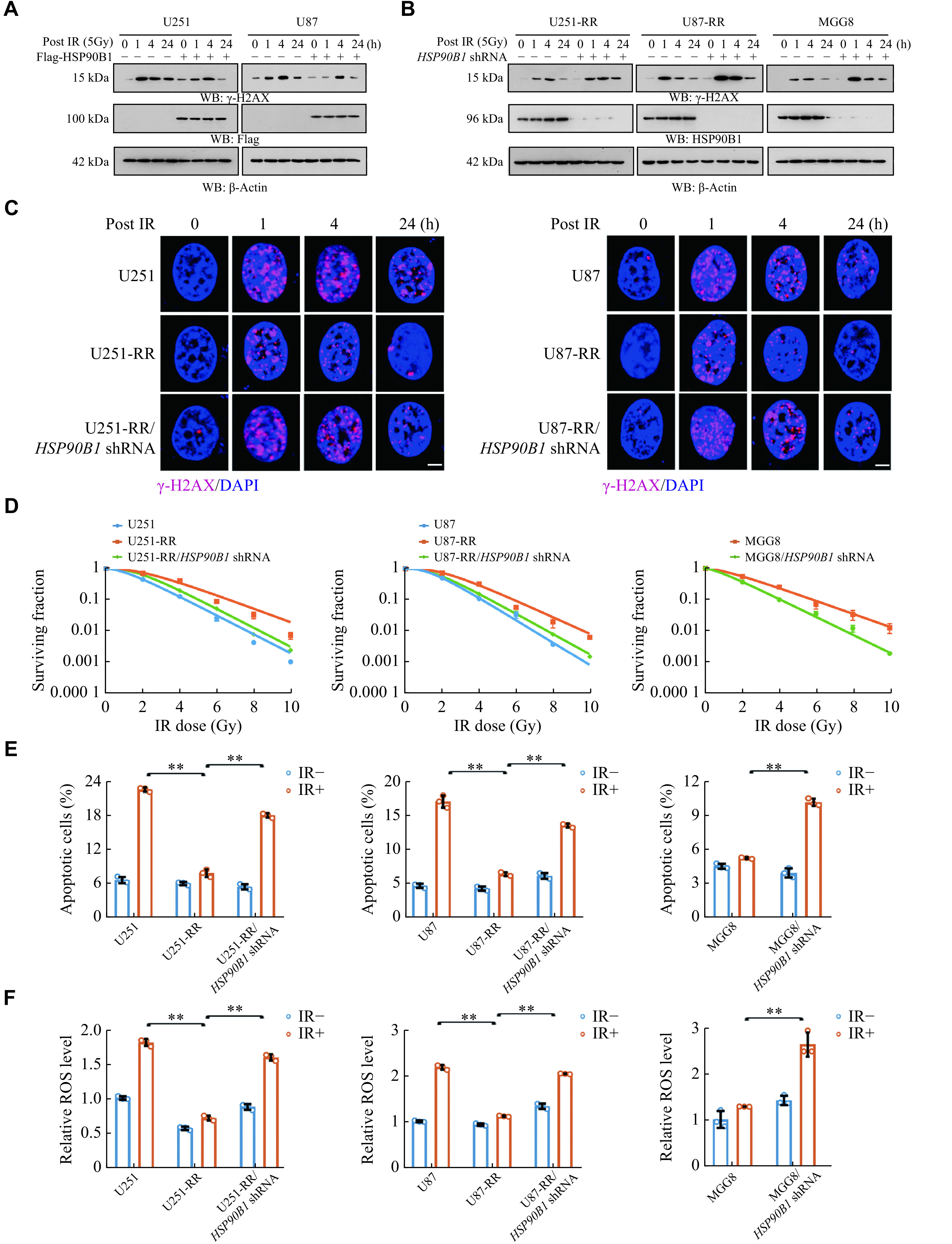
HSP90B1 inhibits the sensitivity of GBM cells to radiation therapy.

The expression levels of HSP90B1 were increased in stably radioresistant GBM cells, compared with their parental counterparts ( ***Supplementary Fig. 3A ***, available online). This increase was also recapitulated in response to short-term IR treatment ( ***Supplementary Fig. 3B ***, available online). Overexpressing Flag-HSP90B1 in the parental GBM cell lines accelerated repair of IR-induced DNA damage as evidenced by reduced levels of γ-H2AX ( ***Fig. 2A***). On the contrary, depleting *HSP90B1* in the radioresistant GBM cell lines, as well as MGG8, a type of GSCs that is innately resistance to radiotherapy, impaired repair of IR-induced DNA damage as evidenced by prolonged levels of γ-H2AX ( ***Fig. 2B***) and longer-lived γ-H2AX positive foci ( ***Fig. 2C*** and ***Supplementary Fig. 3C*** [available online]). In line with these observations, the depletion of *HSP90B1* diminished survival ability of radioresistant cells upon radiation challenges ( ***Fig. 2D*** and ***Supplementary Fig. 3D*** [available online]), to a similar extent to that of parental cell lines.

Compared with parental cells, radioresistant GBM cells showed a limited increase of apoptotic levels after radiation treatment; these effects were abrogated when HSP90B1 was depleted in these cells ( ***Fig. 2E***, ***Supplementary Fig. 3E*** and ***3F*** [available online]). IR induces ROS generation, which is a key mediator of radiation-induced cell death. Consistently, the knockdown of *HSP90B1* in radioresistant GBM cells resulted in increased levels of ROS ( ***Fig. 2F***). Taken together, these results suggested that HSP90B1 reduced the sensitivity of GBM cells to radiation.

### HSP90B1 enhances the glycolytic capacity of GBM cells

**Figure 3:**
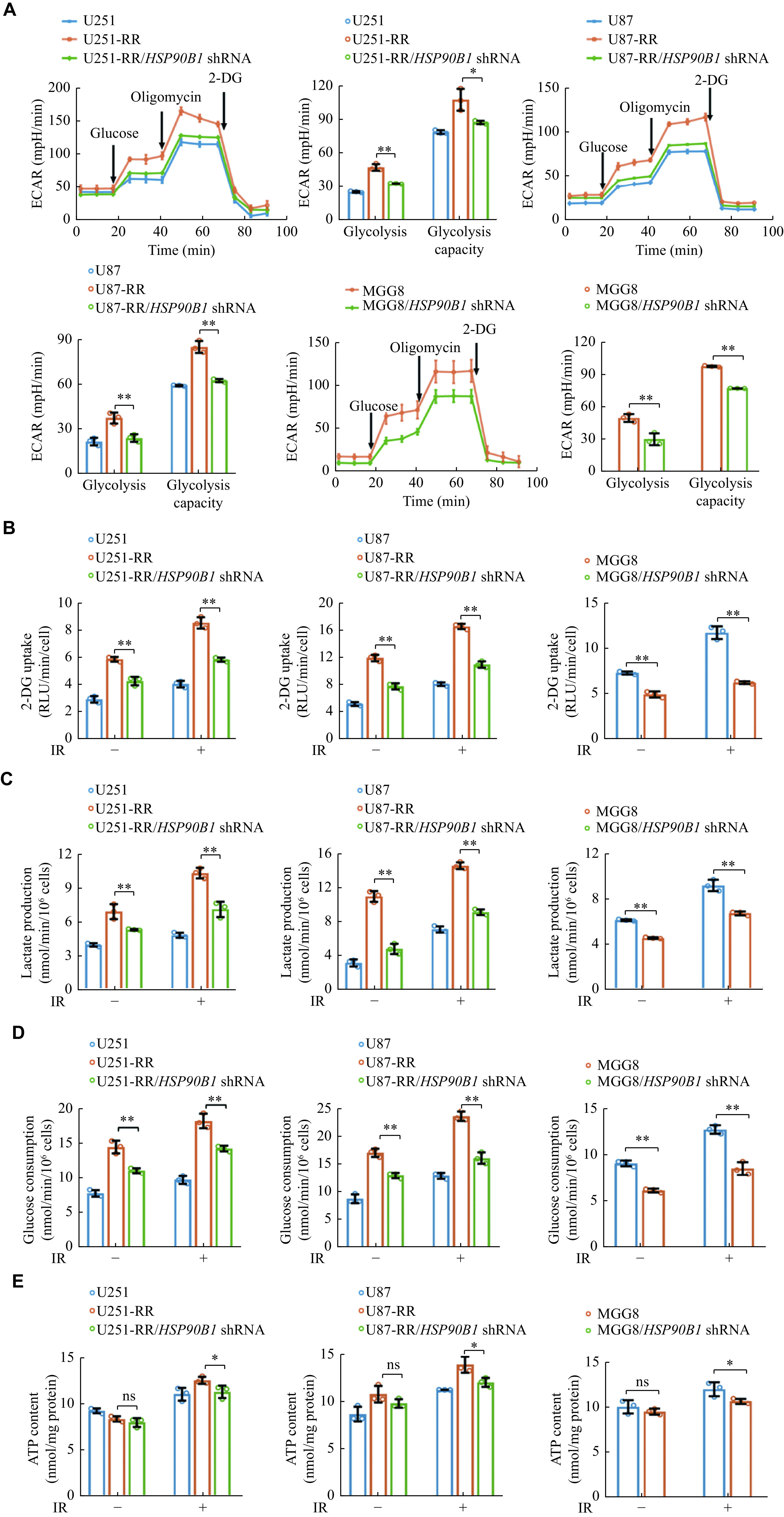
HSP90B1 enhances the glycolytic capacity of GBM cells.

To explore the pathways modulating resistance to GBM radiotherapy, we analyzed our RNA-seq data. The gene set variation analysis of differentially enriched pathways revealed that pathways including protein secretion, hypoxia, apical junction, glycolysis and coagulation were significantly upregulated in RR cells, compared with parental GBM cells ( ***Supplementary Fig. 4A ***, available online). Given that activated glycolysis is one of the hallmarks of cancer cells, including GBMs, we therefore focused our analysis on glycolytic activity. The gene set enrichment analysis confirmed that the glycolytic pathway was much activated in RR GBMs ( ***Supplementary Fig. 4B ***, available online). ECAR analyses revealed that the depletion of *HSP90B1* remarkably inhibited the rate and capacity of glycolysis in U251-RR, U87-RR, and MGG8 ( ***Fig. 3A ***), indicating that the glycolytic activity of GBMs was closely related to HSP90B1 expression. In line with these findings, we detected a decreased glucose uptake ( ***Fig. 3B***), lactate production ( ***Fig. 3C***), and glucose consumption ( ***Fig. 3D***) in *HSP90B1*-depleted U251-RR, U87-RR, and MGG8 cells. We also measured whether HSP90B1 mediated ATP levels, and found that the depletion of *HSP90B1* resulted in reduced levels of ATP when radioresistant cells received irradiation ( ***Fig. 3E***). These results collectively suggested that HSP90B1 promoted glycolytic activity in GBMs.

### HSP90B1 promotes plasma membrane translocation of GLUT1 in GBM cells

**Figure 4:**
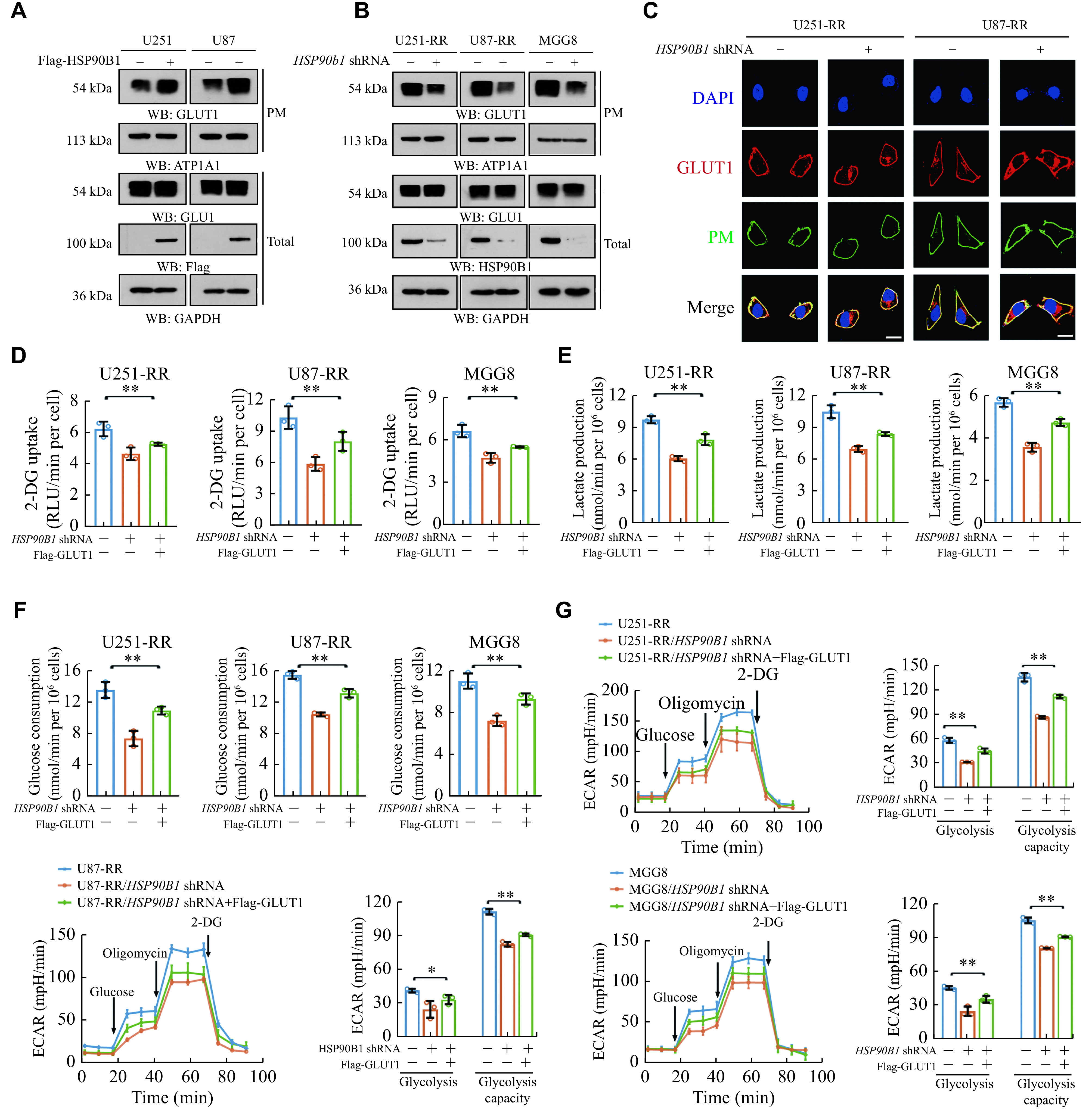
HSP90B1 promotes plasma membrane translocation of GLUT1 in GBM cells.

Glucose has been found to be transported from the extracellular space into the cytoplasm by GLUT1 in cancer cells ^[ 11] ^. No correlation of expression levels between GLUT1 and HSP90B1 was observed in databases ( ***Supplementary Fig. 5A ***, available online) including the TCGA, Gravendeel, and Oh database ^[ 22– 23] ^, suggesting that HSP90B1 may not affect the general expression of GLUT1. Previous reports showed that the activity of GLUT1 was upregulated upon localization to the plasma membrane ^[ 24] ^. To confirm whether HSP90B1 regulates the plasma membrane localization of GLUT1, we isolated cellular fractions. Cell fractionation analysis confirmed that U251 and U87 cells overexpressed with Flag-HSP90B1 demonstrated higher levels of endogenous GLUT1 in the plasma membrane fraction without affecting global GLUT1 protein levels ( ***Fig. 4A***). The depletion of *HSP90B1* in U251-RR, U87-RR, and MGG8 showed similar results ( ***Fig. 4B***). These observations were recapitulated by immunofluorescence analyses ( ***Fig. 4C***).

We next determined whether HSP90B1 regulated glycolytic activity through promoting plasma membrane localization of GLUT1. Simultaneous knockdown of *HSP90B1* and *GLUT1* ( ***Supplementary Fig. 5B ***, available online) significantly inhibited glucose uptake ( ***Supplementary Fig. 5C ***, available online), lactate production ( ***Supplementary Fig. 5D ***, available online), and glucose consumption ( ***Supplementary Fig. 5E ***, available online) in GBM cells. ECAR further confirmed that double depletion of *HSP90B1* and *GLUT1* largely reduced glycolytic rate and capacity ( ***Supplementary Fig. 5F ***, available online). Finally, we tested whether forced expression of GLUT1 ( ***Supplementary Fig. 5G ***, available online) restored *HSP90B1* depletion-reduced glycolytic activities. Overexpressing Flag-GLUT1 in *HSP90B1*knockdown radioresistant cells partially recovered glucose uptake ( ***Fig. 4D***), lactate production ( ***Fig. 4E***), glucose consumption ( ***Fig. 4F***), and glycolytic capacity ( ***Fig. 4G***). These results together showed that HSP90B1 enhanced glycolytic capacity by promoting the plasma membrane localization of GLUT1.

### *HSP90B1*knockdown combined with irradiation improves the survival of mice bearing glioblastoma

**Figure 5:**
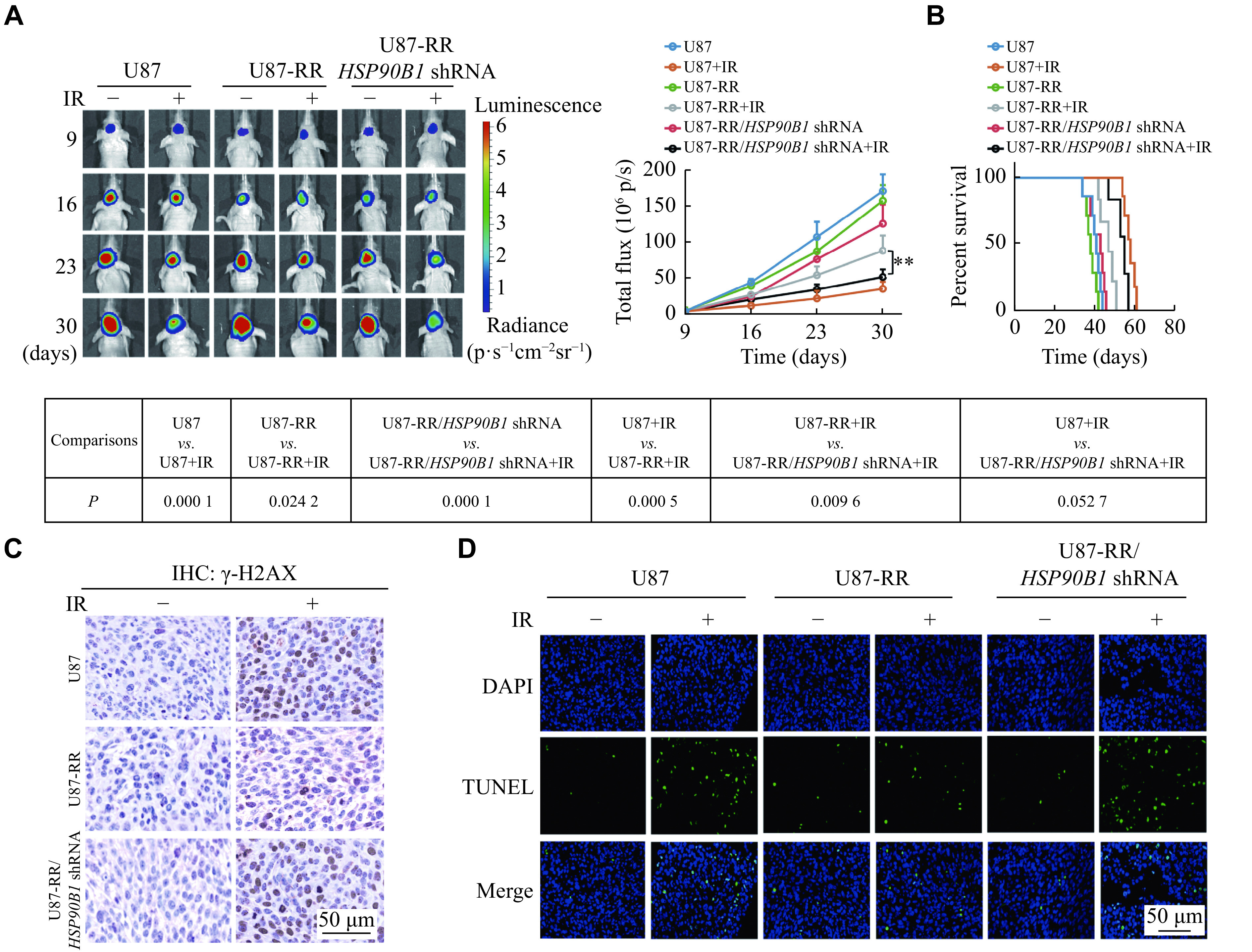
*HSP90B1* knockdown combined with IR improves the survival of mice bearing GBM.

To investigate the functions of HSP90B1 *in vivo*, we injected GBM cells into the brains of nude mice. Five grays of radiation were given for three consecutive days after injection on days 17, 18, and 19 ( ***Supplementary Fig. 6A ***, available online). Bioluminescence imaging showed that the knockdown of *HSP90B1* in combination with radiotherapy remarkably inhibited brain tumor growth ( ***Fig. 5A*** and ***Supplementary Fig. 6B*** [available online]), which was also accompanied by a considerably longer survival time ( ***Fig. 5B*** and ***Supplementary Fig. 6C*** [available online]). Immunohistochemical analysis showed that γ-H2AX levels were greatly reduced in U87-RR after irradiation, compared with U87, whereas knockdown of *HSP90B1* in U87-RR restored γ-H2AX expression ( ***Fig. 5C***). Similar results were shown in the MGG8 GSC group ( ***Supplementary Fig. 6D ***, available online). The TUNEL assay also revealed that the IR-induced apoptotic effects were largely enhanced by *HSP90B1* knockdown ( ***Fig. 5D*** and ***Supplementary Fig. 6E*** [available online]). Collectively, these data suggested that synergistic knockdown of *HSP90B1* enhanced the inhibitory effect of IR on GBM tumor growth.

## Discussion

In the present study, we uncovered that HSP90B1 could modulate the membrane localization of GLUT1, leading to the activation of glycolysis and radiotherapy resistance in GBMs. The analysis of transcriptomic data between radiosensitive and radioresistant GBM samples showed that HSP90B1 and the glycolytic pathway were significantly upregulated in RR GBM cells. Further functional studies revealed that the elevated HSP90B1 promoted the localization of GLUT1 in the plasma membrane, which in turn promoted proliferation and tumorigenesis in GBM cells.

HSP90B1 is a highly conserved molecular chaperone that usually binds to other copolymers and physiologically assists in membrane or secretory protein folding and assembly as well as maintains endoplasmic reticulum homeostasis ^[ 25] ^. It has been shown that HSP90B1 overexpression is associated with an increased malignancy in a variety of cancers, including esophageal ^[ 26] ^, colorectal ^[ 27] ^, breast ^[ 28]^ and lung cancers ^[ 29] ^. The HSP90B1 elevation is often accompanied by alterations in key molecules involved in tumor cell proliferation, invasion and apoptosis ^[ 30] ^, indicating that HSP90B1 has a significant impact on cancer progression and resistance to treatment. In gliomas, the HSP90B1 overexpression was also associated with the increased tumor aggressiveness and the worsening of clinical outcomes ^[ 31] ^, implying a role for HSP90B1 in the development of GBMs. In line with this, by exploring the glioma public database, we observed that HSP90B1 was most highly expressed in the most malignant mesenchymal subtype gliomas, and that patients with higher HSP90B1 expression had a poorer prognosis. More importantly, functional investigations, including animal studies, showed that GBM cells with a high HSP90B1 expression level had a reduced sensitivity to radiotherapy, and the knockdown of *HSP90B1*in RR GBM cells re-evoked radiation-induced ROS production and apoptosis to sensitize GBM cells to radiation therapy.

Irradiation can cause alterations in tumor metabolism. Glucose metabolism, the main source of cellular energy in the body, is also stimulated by irradiation ^[ 32] ^. Glycolysis is a process that begins with the breakdown of glucose to pyruvate and is characterized by the increased glucose uptake and lactate production, ultimately generating ATP. Studies have shown that glycolysis is closely associated with tumor radiation therapy resistance ^[ 33] ^, and clinically, one of the main reasons for radiation therapy failure is the elevated levels of glycolysis. AKT plays a critical role in cell survival and apoptosis, and AKT-mediated alterations in cellular glycolytic pathways were reported to confer radioresistance to tumor cells, when they were exposed to radiation for prolonged periods of time ^[ 34] ^. GBM cells were found to show the upregulation of hypoxia-inducible factor 1 and pyruvate dehydrogenase kinase 1 in response to insufficient blood supply, leading to an increased glycolysis ^[ 35] ^. Moreover, one study showed that the elevated rates of glycolysis promoted the rejoining of radiation-induced DNA strand breaks by activating the non-homologous end joining and homologous recombination pathways, thereby reducing radiation-induced genetic damage in cancer cells ^[ 36] ^. Given that changes in some key molecules in glucose metabolism can affect the efficiency of radiotherapy, targeting glycolysis may be an important part to improve cancer treatment efficacy ^[ 37] ^. Consistent with this finding, we demonstrated the elevated levels of glycolysis in radioresistant GBMs, with GLUT1 being the first rate-limiting step in glycolysis. Using cellular fraction analysis, we observed that HSP90B1 promoted the membrane localization of GLUT1 and subsequently led to the increased glucose uptake and lactate production. However, there was no obvious change in ATP production in radioresistant GBM cells with HSP90B1 knockdown when unirradiated, probably because cells, when deficient in nutrients, maximize ATP output to meet survival needs. When irradiated, the inefficient ATP synthesis following the Warburg effect is not sufficient to maintain the energy requirements of the cells.

In conclusion, our results suggest that a decrease in HSP90B1 induces a reduction in GLUT1 plasma membrane localization and a decline in glycolytic activity, ultimately leading to a desensitization to radiotherapy. These findings highlight the importance of HSP90B1 in the progression of GBM radioresistance. Targeting HSP90B1 and the related glycolytic pathways may provide therapeutic benefits for patients with intrinsic and acquired radioresistance.
